# Financial Incentives for Promoting Participation in a School-Based Parenting Program in Low-Income Communities

**DOI:** 10.1007/s11121-019-0977-y

**Published:** 2019-01-15

**Authors:** Deborah Gross, Amie F. Bettencourt

**Affiliations:** 1 Johns Hopkins School of Nursing, Baltimore, USA; 2 Johns Hopkins School of Medicine, Baltimore, USA

**Keywords:** Financial incentives, Parenting, Early childhood, Low-income families, Chicago Parent Program

## Abstract

Although financial incentives are a well-accepted strategy for raising parent participation rates in prevention studies, they are rarely employed in practice due to concerns about their ethics, sustainability, and public acceptability. We sought to address these common concerns in the context of a larger prevention study using financial incentives to boost parent participation in a group-based parenting program implemented in an urban school district. We examined the extent to which the financial incentives delivered via bank debit cards ($15 for attending weekly group sessions, $5 for completing weekly practice assignments) motivated parents to enroll in the program and were associated with higher attendance and practice completion but poorer participation quality in group sessions, and how parents used the extra cash. Over 3 years, 67.4% (*n* = 372) of eligible families enrolled in a parenting program called the Chicago Parent Program. Most parents were African American (68%) or Latinx (24%); 67% reported annual household incomes < $20,000. Although 71.2% of parents reported that the financial incentives motivated their enrollment, the most important motivators pertained to wanting to be a better parent. Parents citing incentives as motivating their enrollment had higher attendance than those who did not (*p* = .01). Quality of parent participation was high and unrelated to whether financial incentives motivated enrollment. Parents reported using the extra cash to purchase items for their children (92%) and groceries (56%). Results suggest that financial incentives targeting low-income families of young children may improve parent participation rates without diminishing their intrinsic motivation to improve their parenting.

The benefits of preventive parenting interventions for strengthening parenting skills and improving children’s behavioral well-being are well documented (National Academies of Sciences, Engineering, and Medicine 2016). When targeted toward parents of younger children, these types of parent-focused interventions have led to significant and sustained improvements in parenting behavior and confidence, decreases in child conduct problems, improved academic outcomes, and reductions in child maltreatment rates (Brotman et al. 2016; Comer et al. 2013; Prinz et al. 2009). However, it is also well documented that participation rates in preventive parenting interventions among families struggling with economic and psychosocial disadvantage are low (Gonzalez et al. 2018). Typically, over one third of low-income parents who enroll in group-based parenting programs never attend and among those who do attend, average attendance rates hover around 50% of sessions (Baker et al. 2011; Garvey et al. 2006). Yet, parents challenged by chronic economic hardship, whose children face the greatest odds for acquiring a range of social, emotional, behavioral, and academic problems, are typically the primary targets of early intervention efforts (Shonkoff et al. 2012).

Low participation rates in evidence-based parenting programs are an important problem. It reduces program reach and impact and inflates the cost of program delivery, making them unsustainable in low-resource settings. Gross et al. (2011) found that when 15 parents sign up for a parenting program but only one attends, implementation costs (e.g., staff time, materials, refreshments, space) can exceed $900 per parent per session (in 2009 dollars) compared to $88 per parent per session when all 15 parents attend. As a result, agencies serving low-income families may be reluctant to offer evidence-based parenting programs because the cost for such low yield is difficult to justify when resources are limited.

Low parent attendance does not necessarily mean parents do not want help with their parenting. The most common reasons for non-attendance in parenting programs have typically pertained to limited time, logistical barriers (e.g., lack of transportation or childcare), or scheduling conflicts rather than disinterest in obtaining support with their parenting (Garvey et al. 2006). A national survey of parents of young children found that across all income groups, the majority of parents wished they had more information about how to be a better parent and over 80% agreed that good parenting can be learned (Lerner and Nightingale 2016). However, like many other health behavior changes people say they would like to make, such as smoking cessation and weight loss, changing parenting behavior can be challenging.

## Financial Incentives for Improving Parent Participation Rates

One strategy for increasing participation rates in evidence-based parenting programs is offering financial incentives contingent on participation. Based on the principles of behavioral economics, financial incentives can help sway people’s decision when faced with competing demands (Haff et al. 2015; Rice 2013). The theory relies on people’s tendencies to focus on the present and discount the future. For example, a parent with 2 h to spare is faced with the option of using that time to attend a parenting program that could provide future benefits for the child and family, complete some important errands, or take a much-needed break to relax. According to behavioral economics, a financial incentive contingent on attending the parent group may motivate the parent to invest in their child’s future now by participating in the program and completing their errands or relaxing at another time. However, money alone may not be sufficient. If the incentive is too small, too delayed, or linked to a behavior the parent does not value or perceives to be outside their control, it is unlikely to be effective (Volpp et al. 2011). Thus, effective incentive programs need to be strategic and meaningful to the targeted recipients.

A few studies have tested the effects of financial incentives on parent participation, though results have been mixed. For example, Heinrichs (2006) tested the effect of financial incentives on parent enrollment and attendance in a 4-week parenting program in German preschools serving low-income families. Families in preschools randomized to receive financial incentives could earn 15€ (approximately $20) for attending each of the four 2-h sessions and 5€ (approximately $7) for participating in four 15-min individual phone sessions. Bonus incentives were provided for perfect attendance and completing all phone sessions (maximum payment 110€ or $145). In this study, financial incentives led to a 76% increase in parent enrollment and 30% increase in attendance rates over the no-incentive condition.

In contrast, Dumas et al. (2010) found no effects of financial incentives for increasing parent attendance in an 8-week parenting program, though incentives did increase the number of parents who signed up for the parenting program, particularly parents who were more economically disadvantaged. The fact that parents were more likely to sign up but not attend the 2-h parenting groups may have been due to the small incentive amount or the complex implementation strategy. Specifically, parents received only $3 for attending the first session (or $1.50/h of their time, not including their travel time or other expenses associated with attendance). If they attended all eight sessions, their average incentive was $8.50 per 2-h session (maximum payment $68), which may have still been too small for the time and effort involved. Moreover, the use of a graduated payment schedule (i.e., $3 per session for attending two group sessions; $6 per session for attending two more sessions; $10 each for attending the next two session; $15 each for attending the last two sessions) may have introduced too much complexity or confusion and demoralization when they did not receive as much money as they had expected.

Neither study provided a clear rationale for how the incentive amount, timing, or strategy was determined. In general, there is limited published evidence supporting best practices with regard to the most effective ways to structure financial incentives (i.e., magnitude, format, behavior best suited for incentives, types of individuals who will most benefit) (Giles et al. 2014). Indeed, in explaining why larger incentive amounts were not used in their study, Dumas et al. (2010) suggest that sensitivity to what most community agencies can afford played a role in their decision to keep the incentive amount low rather than what amount or incentive structure would adequately motivate behavior change in the consumer.

It is important to note that financial incentives are a commonly used and well-accepted strategy for recruiting and retaining participants in prevention studies (Grady et al. 2005). However, they are rarely employed in practice, in part because of their low acceptability outside of a research context. For example, when Fast Track (Conduct Problems Prevention Research Group 2011), an empirically supported program for reducing conduct problems in at-risk adolescents, was disseminated to the local community, school officials prohibited the use of cash incentives for parent attendance that were used in the original trial. The result was a steep decline in parent participation (Dodge 2009).

A common concern is that incentives undermine people’s intrinsic motivation to engage in healthy behavior (Halpern et al. 2009; Ryan and Deci 2000); since people will be motivated by the money, it is presumed that any behavior change will not be sustained. Targeting financial incentives to economically disadvantaged individuals triggers other biases including concerns that the money will not be used “responsibly” (e.g., used for cigarettes, alcohol, illegal substances), that it will create a “culture of entitlement” in which people will come to expect rewards for engaging in responsible behavior, or that financial incentives discriminate against those who engage in healthy behavior without being paid to do so (Giles et al. 2016; Kenny 2015).

There is little evidence to support these concerns. Research shows that low-income parents are more likely to use supplemental income to pay down debt, purchase basic necessities, and invest in their children. For example, the federal Earned Income Tax Credit (EITC) program, which provides working poor families tax refund credits, was created with bipartisan Congressional support to incentivize employment. It has also been linked to a range of positive health outcomes including better maternal-infant health, improved school outcomes, higher college enrollment, and increased earnings among the children of the program’s beneficiaries (Marr et al. 2015). Research on how EITC funds are used shows that most low-income parents spend their extra money on two types of activities: (a) those designed to move their families out of poverty, such as paying off debt, paying bills, and investing in their future by building personal assets and obtaining additional education or training and (b) on rewards or treats for their children (e.g., new clothes, taking them out to eat) and items for their homes (e.g., furniture, household appliances) (Duncan et al. 2007; Mammen and Lawrence 2006; Romich and Weisner 2000; Sykes et al. 2015).

In sum, the evidence to date suggests that financial incentives can be effective for improving health behavior and child outcomes and that low-income parents use the supplemental cash to support their families. However, societal concerns related to the ethics and morality of financial incentives persist. This non-experimental study describes the impact of one financial incentive program designed to boost parents’ enrollment and participation in an evidence-based parenting skills program implemented in an urban school district serving a large population of low-income families. The ChiPP Project was a 3-year partnership with Baltimore City Public Schools to implement the Chicago Parent Program in 12 Title I schools with pre-kindergarten (pre-K) programs serving over 90% low-income students. Based on previous experience, the school principals and district leaders anticipated very low participation in a 12-session program so they were intrigued by the addition of the financial incentive to support parent participation.

The current study focuses on the financial incentives that were used to motivate parent enrollment and participation in the ChiPP Project. Specifically, we address four questions pertaining to the ethical and moral concerns associated with using financial incentives in low-income populations:

1. *To what extent did the financial incentives motivate parents to enroll in the parenting program?* We were particularly interested in the importance of the financial incentives in motivating parents’ decisions to enroll in the ChiPP Project relative to other possible reasons unrelated to the extra money (e.g., parenting support, encouraged by a teacher to sign up). This question was designed to address the concern that parents’ primary interest in signing up for the program was the opportunity to earn extra cash.
2. *What was the relationship between the perceived importance of the incentives and rate of attendance and practice assignment completion?* The ChiPP Project did not employ an experimental design that would allow us to directly test the effect of the incentives on increasing enrollment and participation (i.e., parent attendance and completion of weekly skill-building practice assignments). Nonetheless, we examined the association between parents’ perceptions of the importance of the financial incentives and their subsequent participation. Consistent with the theory, we hypothesized that parents who perceived the financial incentives to be an important motivator in their decision to enroll would (a) attend more parent group sessions and (b) complete more weekly skill-building practice assignments.
3. *Did financial incentives diminish the quality of parents’ participation during the parent group sessions?* Given ethical concerns that financial incentives could diminish parents’ intrinsic motivations to actively engage in the parent group sessions, group leaders assessed the extent to which each of the parents was actively engaged in the group sessions. If the incentives had a negative effect on parents’ intrinsic motivations to strengthen their parenting, we would expect to see a low level of parent engagement during the parent group sessions.
4. *How did parents report using the extra cash received from the financial incentive program?* This question was designed to address public concerns that low-income parents might not use the extra cash for good (e.g., to invest in their children, pay bills, purchase basic necessities). To address this concern, parents reported how they used the extra cash.

## Methods

### Participants

Study participants were parents, grandparents, or legal guardians (hereafter referred to as “parents”) of pre-K students attending 12 Title I elementary schools in Baltimore City Public Schools. All 12 schools served a large population of racial/ethnic minority and economically disadvantaged students (> 90% qualified for free or reduced lunch program; > 95% racial/ethnic minority). Participants were recruited through a combination of flyers and school information sessions highlighting the parenting program and the financial incentives (i.e., flyers included the headline “Earn while you learn”). The study was reviewed and approved by the Johns Hopkins University and Baltimore City Public School Institutional Review Boards.

All English- or Spanish-speaking parents of a 4-year-old pre-K student were eligible to participate in the ChiPP Project with the goal of enrolling 15 parents per parent group (estimated pre-K population = 534 students). Over the course of the 3-year study, 372 parents of 380 4-year-old children enrolled in the ChiPP Project, defined as completing the consent form and baseline measures. In some cases when a full group of 15 parents had not been enrolled, school staff requested to offer the program to parents of 4-year-old children enrolled in their Head Start classrooms. As a result, 20 participants were from Head Start. Based only on the pre-K population, 67.4% of the eligible population enrolled (i.e., 360 of 534 eligible pre-K students).

### The ChiPP Project Intervention

The ChiPP Project was initiated in 2014. The primary goals of the initiative were to strengthen parenting skills and improve students’ social-behavioral readiness to learn by kindergarten through parents’ participation in the Chicago Parent Program (CPP) (Bettencourt et al. 2018). The CPP is a 12-session evidence-based parenting program designed in collaboration with an advisory board of African American and Latinx parents raising young children in low-income urban communities (Gross et al. 2007; Gross et al. 2014). Previous research has demonstrated that the CPP improves positive parenting behavior and confidence and reduces corporal punishment and child behavior problems in low-income families and families of color (Breitenstein et al. 2012; Gross et al. 2009, Gross et al. in press).

CPP content and structure is based on social learning theory and the coercive process model (Patterson 1982) which highlights how parents model and reinforce their children’s positive as well as aversive behavior. The program is also guided by concepts relevant to attachment theory and the essential role of stable, responsive, and nurturing adults in young children’s lives (Biglan 2015; Bowlby 1982). Parents learn parenting strategies using video vignettes, group discussion and problem-solving, role play and group activities, brief handouts, and weekly skill-building practice assignments. Group leaders use a detailed manual for guiding the parent group discussions and activities and helping parents tailor strategies to their parenting goals and values. All groups are led by two trained group leaders who have at least a high school diploma. The first four sessions focus on helping parents clarify their values and goals for their children and using strategies for building a positive relationship. The second 4 weeks focus on helping parents set clear expectations for their children based on their values and parenting goals and using effective limit setting strategies to achieve those goals. Three subsequent sessions center on stress management; problem-solving skills; and reviewing what has been learned, what is working, and what parents still need to work on. A 12th “booster” session is offered approximately 1 month later to discuss how parents are managing without the ongoing support of the parent group. Food and childcare are provided during all parent group sessions.

All parent groups were offered at the school in English or Spanish, usually in the mornings after children were dropped off at school, or in the evenings, based on school leadership preference. Over the course of the 3 years, 22 CPP groups were conducted in English and 6 CPP groups were conducted in Spanish. Of the 23 group leaders who led CPP groups, 9 (39.1%) were African American, 11 (47.8%) were White, 1 (4.4%) was Asian, and 1 (4.4%) was Latinx. Four (17.4%) were bilingual in Spanish and English. The majority of group leaders (69.6%) were employed by the school. Eighteen (78.3%) CPP group leaders held a graduate degree in a mental health field while the remainder were paraprofessionals with experience working with families. CPP group leaders helped to enroll parents at their respective schools by attending parent meetings and presenting the program to the teachers.

In a previous paper (Bettencourt et al. 2018), we reported that parent participation rates in the ChiPP Project were strong; 78.5% of the 372 enrolled parents attended at least one parent group session. Among those who enrolled but never attended (*n* = 80), 52.5% (*n* = 42) indicated that non-attendance was due to getting a new job or changes in their work schedule. Other reasons for non-attendance were health issues (*n* = 5; 6.3%), life stressors (*n* = 2; 2.5%), childcare issues (*n* = 2; 2.5%), schedule conflicts (*n* = 3, 3.8%), and changing schools (*n* = 4; 5.0%). Among those who attended at least one session, average attendance was 8 of 12 (67%) sessions and parents completed on average 6 of 10 (66%) practice assignments.

Children’s behavior problems also improved (Bettencourt et al. 2018). Although the ChiPP Project was available to all pre-K parents, 40% of the children of enrolled parents had behavior problems in the clinical range at baseline on the Eyberg Child Behavior Inventory (ECBI; Eyberg and Pincus 1999). At post-intervention, there was a 37% decrease in the number of children with behavior problems in the clinical range and mean child behavior problem scores dropped significantly (*p* < .001). These data show that although the parents received financial incentives for their participation, parents appeared to apply what they were learning and there was a substantial reduction in their children’s behavior problems.

### Financial Incentive Program

At enrollment, each parent received a bank debit card, which was used to electronically distribute incentives to parents. Group leaders submitted attendance and practice assignment completion records to the coordinator after the group session and funds were electronically transferred onto parents’ bank debit cards within 48 h of the group session. Debit cards could be used to purchase goods or services from any business that accepted bank debit cards or to withdraw cash from an automated teller machine (ATM). There was no usage fee unless parents used the card to withdraw cash from an ATM. Lost debit cards could be replaced.

For each weekly 2-h parent group session attended, $15 was loaded electronically onto their debit card. Parents needed to attend at least 75% of the 2-h session to receive the financial incentive. For each weekly skill-building practice assignment submitted, parents received another $5 on their debit card. Parents could earn a maximum of $230 for attending all 12 parent group sessions and submitting all 10 skill-building practice assignments. Bonuses were not given for perfect attendance as we did not want to penalize parents who could not attend if circumstances beyond their control prevented attendance (e.g., parent or child was ill, employer changed parent’s work schedule). The selected magnitude of the incentives used was guided by prior research on the opportunity costs to parents for attending 2-h weekly CPP groups (Gross et al. 2011). By linking the incentive amount to parent opportunity costs, which we assumed to be comparable to a minimum wage job, the incentive was intended to honor their time, be of sufficient magnitude as to provide extra cash to supplement their existing incomes, but not be so large as to be coercive. We also wanted to link the incentive to a discrete behavior (i.e., attendance in the 2-h parent group, submitting practice assignment checklists) rather than an outcome (e.g., improved behavior) based on research demonstrating that incentives tied to discrete behaviors tend to be more effective than complex behavior change (Kane et al. 2004; Sutherland et al. 2008). The financial incentive was structured to provide guaranteed payments for attendance and practice assignment completion rather than a lottery-based incentive (i.e., only some attendees receive an incentive based on random drawings) or a debit model (i.e., removing cash for non-attendance) to provide immediate positive reinforcement for each incentivized behavior.

### Variables and Measures

#### Motivations for Enrolling in the Parenting Program

The Parent Motivation Form (Gross et al. 2001) was used to measure parents’/guardians’ motivations for enrolling in the parent program. This checklist includes a list of 13 reasons why parents might want to attend a parenting program including parenting needs (e.g., “I am always looking for ways to be a better parent”), encouragement from others (e.g., “My child’s teacher recommended that I participate in this program”), social interests (e.g., “I would like the chance to talk with other parents with young children”), and financial incentives (e.g., “I would like the extra money for attending the parent group”). At baseline, participants were asked to first indicate whether each reason listed was an important motivator in their decision to enroll in the groups (yes/no) and to then select from the list the one reason that was “most important” in their decision to enroll.

#### Program Attendance and Practice Assignment Completion

Group leaders recorded participant attendance at each weekly CPP session. Participant attendance rate was calculated as the proportion of the 12 CPP sessions they attended. The program also included 10 weekly skill-building practice assignments in which parents were instructed to practice the new skills during the week and submit a brief checklist documenting their practice. Participant’s practice assignment completion rate reflected the percentage of the 10 CPP assignment checklists completed over the course of the program. Within 24 h of each parent group session, the group leader electronically submitted the record of attendance and practice assignment completion for that session to the ChiPP Project Director.

#### Quality of Program Participation

The Engagement Form was used to measure the quality of parents’ participation in the group sessions (Garvey et al. 2006). This seven-item measure, completed by the group leaders between the 11th and 12th (booster) sessions, assesses the extent to which each parent had actively participated in group sessions over the course of the 11 weeks. Active participation or engagement in groups was defined as the extent to which the parent seemed to pay attention to video vignettes, actively participated in group discussions, was supportive to other members of the group, was open to trying new strategies, and correctly applied program principles outside of the group. Items were rated from 1 (*Not at all*) to 4 (*Most of the time*) and summed*.* Engagement forms were completed only for parents who attended at least one parent group session. Both CPP group leaders independently completed engagement ratings on each parent; the two sets of scores were averaged for each parent. Group leaders were not informed of the study hypotheses. Cronbach’s alpha reliability was .83 and inter-rater reliability between group leader ratings was .66. Past research demonstrating significant positive associations between engagement scores and changes in ratings of child behavior problems provide evidence of convergent validity (Garvey et al. 2006).

#### Parent-Reported Use of Financial Incentives

After the 11th group session, parents completed a checklist for how they had used their incentives to date. Parents could also add other uses for the money if not listed. To generate this checklist, three focus groups (*n* = 19 parents) were conducted in the first set of parent groups and asked to respond to the following question: “What kinds of things did you use your debit card to pay for?” Similar item purchases were combined (e.g., cleaning items, food, and groceries, were merged into one category called “groceries”). Responses from these 19 parents were used to construct the checklist and are not included in the data reported in the present study.

#### Demographic Background Form

At enrollment, parents completed a demographic background form that included information on the parents’ age, race and ethnicity, educational level, employment status, marital status, and income, and the target child’s sex and age. To understand the extent to which the parents were experiencing material hardships, we also asked them to indicate whether they had experienced one or more of seven hardships in the prior 12 months: (1) unable to pay rent or mortgage, (2) evicted for not paying rent or mortgage, (3) unable to pay their utility bill, (4) had utility services turned off because they were unable to pay the bill, (5) had their phone disconnected because they were unable to pay the bill, (6) had someone in their household who needed to see a doctor or go to the hospital but did not do so because they did not have insurance or could not afford to pay for the health care, and (7) had someone in their household who needed to see a dentist but did not do so because they did not have dental insurance (McLoyd 1990).

### Data Analysis

Although CPP is a group-based intervention, intra-class corrections (ICC) showed minimal association among participants within CPP groups on session attendance (ICC = 0.00), practice completion (ICC = 0.00), and participation quality (ICC = 0.02). Therefore, these data were analyzed at the individual level. All analyses were performed in SPSS version 24 (IBM 2016).

Frequencies were calculated to examine counts and proportions of participants identifying specific motivations for enrolling in the groups and to understand what types of items participants purchased using the incentives. Bivariate analyses were conducted to examine demographic differences among those identifying the incentives as not a motivator, as a motivator but not the most important motivator, and as the most important motivator. Univariate general linear models (GLM) were conducted to examine whether the extent to which parents identified the incentives as motivating at enrollment significantly predicted differences in attendance and practice assignment completion rates. In both analyses, the extent to which the incentives motivated enrollment was treated as a fixed effect. Demographic variables shown to be significantly associated with parents’ perceptions of the money as a motivator and with attendance were included as covariates in the models.

## Results

As shown in Table 1, most parents were African American (68%) or Latinx (24%), were the target child’s mother (78%), headed single-parent households (58%), and had a high school diploma or less (69%). The mean (SD) age of parents was 32.5 (8.6) years. Forty-three percent of the parents worked part- or full-time and 67% reported an annual household income of less than $20,000 per year; 44.6% (*n* = 165) reported two or more financial hardships in the prior 12 months. All of the target children were 4 years old at the time of the study and 46.7% were boys.

**Table 1:** Sample demographics (*N* = 372)

Sample characteristic	*N* (%)	Mean (SD)
Parent age (years)		32.5 (8.6)
Relationship to pre-K child		
Mother	290 (78.0%)	
Father	38 (10.2%)	
Grandparent	29 (7.8%)	
Other adult caregiver	15 (4.0%)	
Parent race/ethnicity		
African American	252 (68.1%)	
Hispanic	89 (24.1%)	
White	18 (4.9%)	
Other	11 (2.9%)	
Education level		
Less than a high school diploma/GED	112 (30.1%)	
High school diploma/GED	142 (38.2%)	
Some postsecondary education	118 (31.7%)	
Marital status		
Married	83 (22.3%)	
Single	214 (57.5%)	
Unmarried, but living with partner	74 (19.9%)	
Annual household income		
Less than $10,000/year	153 (41.1%)	
$10,000–$19,999/year	96 (25.8%)	
$20,000–$39,999/year	78 (21.0%)	
$40,000 or more/year	38 (10.2%)	
Employment status		
Full-time	110 (29.6%)	
Part-time	48 (12.9%)	
School	26 (6.9%)	
Not working	188 (50.5%)	
Hardships in the past 12 months		
Unable to pay rent/mortgage	82 (22.2%)	
Evicted for not paying rent/mortgage	12 (3.2%)	
Unable to pay full gas/electricity bill	141 (37.9%)	
Gas/electricity turned off for nonpayment	46 (12.5%)	
Telephone disconnected for nonpayment	105 (28.2%)	
No medical care obtained due to no insurance or inability to pay	67 (18.1%)	
No dental care obtained due to no insurance or inability to pay	110 (29.7%)	

As shown in Table 2, 71.2% of parents identified the promise of receiving extra money for participating in the groups (for attendance, homework assignment completion, or both) as a motivator for enrolling in the groups. Although most parents identified the financial incentives as *a* motivator for enrolling in the program at their child’s school, only 5.9% identified them as the *most important* motivator for enrolling. The motivations most frequently cited as *most important* in their decision to enroll centered on strengthening their parenting; 34.4% checked “are always looking for ways to be a better parent,” 21.4% checked “wanted to learn ways to better manage their child’s behavior,” and 19.2% checked wanting “to learn better ways to communicate with their child” as the most important motivator for them.

**Table 2:** Parent motivations for enrolling in the ChiPP Project (*N* = 372)

Motivation for enrolling	Important^1^ [*n* (%)]	Most important^2^ [*n* (%)]
Extra money to attend	218 (58.6%)	9 (2.4%)
Extra money for using new parenting skills	250 (67.2%)	13 (3.5%)
Learn better ways to manage my child’s behavior	354 (95.2%)	81 (22.0%)
Learn better ways to communicate with child	352 (94.6%)	71 (19.2%)
Chance to talk with other parents	340 (91.4%)	13 (3.5%)
Free meal while attending	233 (62.6%)	1 (0.3%)
Chance to relax without paying babysitters	228 (61.3%)	6 (1.6%)
Help with disciplining child	263 (70.7%)	36 (9.8%)
Another parent recommended I sign up	91 (24.5%)	0 (0.0%)
My child’s teacher recommended I sign up	158 (42.5%)	0 (0.0%)
The recruiter motivated me to sign up	277 (74.5%)	7 (1.9%)
Always looking for ways to be a better parent	357 (96.0%)	127 (34.4%)
Other	34 (9.1%)	0 (0.0%)

^1^Parents could select multiple reasons as important

^2^Parents could select only one reason as the most important motivator

Parents who identified the money as a motivator tended to have annual household incomes of less than $20,000 per year (*χ*^2^ = 9.596, *p =* .01) compared to those who did not identify the money as a motivator. Among those who identified the money as a motivator for enrolling (*n* = 235), 73.2% had a household income of less than $20,000 per year. Similarly, 81.8% of parents who identified the money as the most important motivator for enrolling (*n* = 22) also reported an annual household income of less than $20,000. In addition, the number of financial hardships that a parent reported experiencing in the past year differentiated those who did and did not identify the money as a motivator (*χ*^2^ = 9.231, *p* = .01). Specifically, 59.1% of parents who indicated that the money was the *most important* motivator (*n* = 22) reported experiencing two or more financial hardships in the past year. In contrast, only 32.7% of those not identifying the extra money as a motivator (*n* = 107) reported two or more financial hardships in the past year.

A univariate GLM was conducted to examine differences in attendance and practice assignment completion rates by the degree to which the financial incentives motivated parent’s enrollment (i.e. *not* a motivator; *a* motivator, but not the most important motivator; the *most important* motivator). Results of the unconditional GLM indicated that the extent to which the financial incentives were a motivator was a significant predictor of program attendance (*F* (2, 369) *=* 4.73, *p* = .01). Those identifying the incentives as *a* motivator (56.2%, *p* = .01) and those identifying the incentives as the *most important* motivator (58.7%, *p* = .08) had higher attendance than participants who did not identify the incentives as a motivator (43.0%). After controlling for income and number of financial hardships experienced in the past year, the extent to which the financial incentives were a motivator remained a significant predictor of attendance (*F* (2, 354) *=* 3.51, *p* = .03), with higher attendance rates found among those who identified the financial incentives as a motivator (55.8%) compared to those not identifying them as a motivator (43.3%, *p* = .01). No statistically significant differences in attendance rates were found between those identifying the financial incentives as the *most important* motivator (58.7%, *p* = .11) and those not identifying them to be a motivator.

We also examined the relationships between the extent to which financial incentives motivated enrollment and weekly practice assignment completion. Results of the unconditional model indicated that the extent to which the incentives were a motivator was a significant predictor of practice assignment completion (*F* (2, 369) *=* 3.36, *p* = .04), with those identifying the incentives as *a* motivator (55.1%, *p* = .01) and those identifying the incentives as *the most important* motivator (55.0%, *p* = .21) completing more practice assignments than participants not identifying incentives as a motivator (42.7%). However, after controlling for income and number of financial hardships experienced in the past year, the extent to which the financial incentives were a motivator was no longer a significant predictor of practice assignment completion (*F* (2, 354) *=* 2.32, *p* = .10).

The quality of parents’ participation in groups as rated by the group leaders was high with a mean of 24.3 (SD = 3.6; possible score range = 7–28). A one-way ANOVA was conducted to examine mean differences in the quality of parents’ participation in the groups based on the extent to which the financial incentives motivated participation. There were no statistically significant differences in the quality of parent’s participation between groups that varied in whether and to what extent participants perceived the financial incentives as motivating participation (*F* (2, 286) *=* 4.24, *p* = .72).

As shown in Table 3, more than half of participants reported using the funds to purchase groceries. Cumulatively, the most frequently reported use of the funds was for items for their children including something nice or fun for them (41.7%), clothes (27.8%), books or school supplies (21.8%), and diapers (13.5%). These data are consistent with parents written comments provided on the checklist, which suggest they had purposefully planned to use the extra money for their children and family:

The class was to help me deal and understand how to deal with kids, so I spent it on them.

Because it was to help improve my child and [my] relationship. Every week that [practice] assignment was done with my child so I used it for her. For participating and doing well.

Saved all the money to buy Christmas gifts but ended up using as family emergency.

I haven’t used any of it yet. I will purchase my grandsons something they can use.

**Table 3:** Frequency counts and proportions for how participants used the financial incentives (*N* = 252)

Item	*n* (%) reporting
Groceries	142 (56.3%)
Something fun/nice for my children	105 (41.7%)
Clothes for my children	70 (27.8%)
Gas	59 (23.4%)
Books or school supplies	55 (21.8%)
Took family or friends out to eat	45 (17.9%)
Medicine	43 (17.1%)
Cash from ATM	42 (16.7%)
Diapers	34 (13.5%)
Have not used it yet; saving	33 (13.1%)
Items for my home (e.g., TV, furniture)	32 (12.7%)
Something fun/nice for myself	28 (11.1%)
Clothes for myself	20 (7.9%)
Phone bill	19 (7.5%)
Utilities, cable, or other bills	16 (6.3%)
Gifts for other people	10 (4.0%)

Parents who enrolled in fall 2014 and parents who did not attend any parent groups did not complete the checklist and are excluded from this analysis

## Discussion

This study evaluating the use of financial incentives to boost parent enrollment and participation in a 12-session preventive parenting intervention was designed to address common societal concerns associated with using financial incentives in low-income populations. The results suggest that although 71% of parents identified the extra cash as a motivator for signing up for the parent program, *the most important* motivators cited by parents pertained to wanting to be a better parent. The top three reasons parents selected as the most important motivation for signing up for the parent program were (1) looking for ways to be a better parent, (2) wanting to learn better ways to manage their child’s behavior, and (3) seeking better ways to communicate with their child. Less than 7% rated the extra cash as the most important motivator. These results demonstrate that parents who struggle economically are like all other parents who seek the best for their children.

Nonetheless, the financial incentives were meaningful to these parents and linked to higher attendance, particularly among families facing more economic disadvantage. Parents who rated the financial incentives as the most important motivator for enrolling in the program had lower incomes and experienced more financial hardships in the prior 12 months. Compared to parents not citing the financial incentives as a motivator, those rating the financial incentives as a motivator had higher attendance rates. These results suggest that the incentives were appealing to the most economically disadvantaged parents and functioned as intended by spurring parents to invest now in their children’s futures by attending the program and learning new skills. In fact, one employed parent reported using her vacation days to attend the parent group.

Although parents who rated the financial incentives as a motivator had higher practice assignment completion rates, that result was no longer statistically significant after controlling for income and number of financial hardships. It is possible that the higher attendance led to more parent exposure to group leaders’ reinforcement of the importance of the practice assignments, which may have been more effective than the $5 incentive. The decision to assign a lower magnitude of incentive to practice assignment completion was due to the fact that the actual practice was based on self-report and could not be verified. Perhaps a higher incentive amount for submitting practice assignment checklists would have resulted in greater differences.

Importantly, the financial incentives did not appear to diminish parents’ intrinsic motivations to actively engage in the parent group sessions. Parent engagement ratings showed that parents were highly engaged in the group sessions. In fact, the parent engagement scores obtained in this study are as high as those reported in previous studies not using financial incentives (Garvey et al. 2006; Gross et al. 2011). These results suggest that the financial incentives did not diminish parents’ engagement in the program. However, it is possible that because group leaders rated parent engagement only once at the end of the program, their ratings may have been biased by the quality of the parents’ participation toward the end of the group.

One reason why the financial incentives may not have diminished participation quality is that the incentivized behavior, attending the parent group, was linked to something that was perceived to be meaningful and beneficial. As one participant wrote, “It started out that the money was the reason I got involved. Then the programs instruction and leaders were the reason I continued to come back.” This comment highlights the ethical importance of matching the incentive to participation in a program that (a) participants value, (b) has been demonstrated to improve health outcomes in the target population (i.e., low-income families, families of color), (c) is open to all parents in the grade, thereby limiting any stigma associated with participation, and (d) is linked to long-term benefits to families and society (Krubiner and Merritt 2017).

Finally, parents reported how they used the extra money. Consistent with prior studies on low-income parents’ use of extra funds received from earned income tax credits (Duncan et al. 2007; Sykes et al. 2015), the parents in this study used their financial incentives to purchase essential goods. The most common purchases participants reported making with their financial incentives were items for their children (92% used the money for children’s clothes, school supplies, and something nice/fun for their children) and groceries (56%). It is important to acknowledge that these results are based on parent self-report and may have been biased by social desirability, as we were unable to corroborate their reports using the bank debit card software. Additional research using verifiable strategies for collecting data on the types of purchases made with the financial incentives is recommended.

Another study limitation is the lack of a control group that did not receive financial incentives for participation. Based on the interests of the schools and funders and the potential for demoralization among economically disadvantaged parents not receiving financial incentives for participation, we did not construct this study as a randomized trial (i.e., the city is nicknamed “Smalltimore” as so many residents are Baltimore natives with childhood friends and relatives throughout the city, raising the likelihood of control group families learning they were not receiving incentives for participation). As a result, we do not know if participation rates were higher or participation quality lower as a result of the financial incentive. Nonetheless, over 67% of eligible parents enrolled in the ChiPP Project, which is more than twice the parent enrollment rate achieved in previous studies using the Chicago Parent Program in similar populations (Garvey et al. 2006; Gross et al. 2011). Future research using a randomized design would better address the extent to which the incentives influenced multiple aspects of parent participation, though those studies would need to be carefully constructed so as to not demoralize families already struggling economically.

The use of a bank debit card system for transferring the incentives to parents had several advantages. Funds could be transferred securely and did not require staff, group leaders, or parents to carry significant sums of cash with them. This was particularly important given that most of the schools were located in high-crime neighborhoods and many parents walked or relied on public transportation to get to and from the school. Another advantage was that participants did not need a bank account to receive or use the debit card, as low-income households are significantly less likely to hold bank accounts (FDIC Unbanked/Underbanked Survey Study Group 2016). Parents also appreciated the fact that the debit cards could be used to purchase goods at any business accepting debit cards. Financial incentives offered in the form of gift cards from select stores may have limited impact if those stores are difficult to access or provide goods families do not need. Finally, unless parents used the debit cards at an ATM, no usage fees were incurred so parents received the full amount earned from their participation. All of these qualities made the bank debit cards an attractive strategy for deploying cash incentives.

However, the debit card system also poses some disadvantages related to cost and sustainability. For example, finding a bank that would provide the debit cards and software for electronically loading the cards each week, budgeting for costs associated with providing the service (i.e., $2.50 fee for each debit card, $0.25 load fee for each new incentive; salary support for a coordinator to manage and load the debit cards), and ensuring group leaders submitted timely and accurate records of parent participation present potential challenges. Although other cash transfer systems could be used (e.g., Venmo through PayPal, Google Pay), all involve fees and management oversight and may require bank accounts. Given the growth of prepaid debit card use among low-income families (FDIC Unbanked/Underbanked Survey Study Group 2016), banks might consider expanding this service to more communities as a way to develop relationships with families that have traditionally shied away from relying on banks. Evaluating the extent to which financial incentives, delivered via bank debit cards and conditioned on pre-specified health behaviors, could also promote financial literacy and savings in low-income households is an important area for future research.

Finally, the financial incentives for participation in the ChiPP Project were supported by local private foundations that understood the need and potential impact of the cash incentives for promoting participation. Indeed, in the years that have followed the official end of the ChiPP Project in 2017, schools and private funders have continued to support its continuation based on the positive outcomes observed from the initiative. However, long-term solutions for sustaining financial incentives to low-income families that benefit their young children are needed. For example, future research centered on the costs and benefits of preventive interventions that include financial incentives could demonstrate whether there is a societal return on investment when evidence-based interventions are coupled with cash incentives to promote participation.

The concept of using cash rewards for encouraging low-income parents to invest in the human capital of their children is not new. Conditional cash transfer (CCT) programs, implemented in over 60 low- and middle-resourced countries, are federally sponsored programs created to (a) reduce poverty (i.e., CCTs can supplement up to 20–30% of family income) and (b) improve children’s long-term health and economic circumstances (i.e., cash supplements are conditioned on families completing pre-determined activities intended to improve their children’s health and developmental wellbeing) (Fiszbein et al. 2009; Lagarde et al. 2007). Large-scale evaluations of CCT programs have demonstrated their positive effects on a range of child health outcomes including child vaccination rates, physical and cognitive development, and school enrollment rates (Carvalho et al. 2014; Fernald et al. 2017). CCT programs are a well-established component of government safety nets for impoverished families in Latin America, Africa, and Asia (Cahyadi et al. 2018).

Although more limited, CCTs have also been tested in the USA. The most well-known CCT demonstration projects have been the Family Rewards programs implemented in New York City and Memphis (Courtin et al. 2018). Results from the Family Rewards studies have shown that the CCTs led to a range of significant improvements including reductions in poverty, material hardship, adolescent aggression, and substance use; more regular dental care; gains in full-time employment, the percent of parents saving money for their children’s education, and participants’ levels of hope (Aber et al. 2016; Miller et al. 2015; Morris et al. 2017). However, CCTs did not lead to improvements in academic outcomes for boys and students who were less academically prepared at study enrollment (Aber et al. 2016). This result suggests that perhaps instituting CCT programs for low-income families earlier in their children’s development may be most beneficial for promoting a strong foundation for school readiness (Duncan et al. 2014).

While the cash incentives used in this study were conditioned on completing specific behaviors designed to encourage parent investment in their children’s futures, the magnitude of incentive was not intended to move families out of poverty. Rather, incentive amounts were designed to supplement supports many of the parents were presumed to already be receiving through publicly funded programs such as Medicaid, Temporary Assistance for Needy Families (TANF), and Supplemental Nutrition Assistance Program (SNAP), with the understanding that many recipients remain cash poor despite these critical safety net programs (Edin and Shaefer 2015). Indeed, less than 25% of TANF funds are distributed to families in the form of cash assistance (Floyd et al. 2017; Schott et al. 2018). Although we did not collect data from families on their income sources or degree of debt, nearly 38% of the families reported being unable to pay their full utility bill at least once over the prior 12 months, and approximately 30% could not see a dentist because they were unable to pay for it.

Consistent with CCT programs that incentivize a range of behaviors promoting parental investments in their young children, initiatives like the ChiPP Project could be one of myriad incentivized prevention programs designed to improve parents’ and children’s health and future economic circumstances (e.g., attending a financial literacy program, increasing pre-K attendance rates). Given that children raised in Baltimore have the lowest odds of escaping poverty relative to children in other large US cities (Chetty and Hendren 2018), a CCT program to improve the health and economic circumstances of its youngest citizens and their families could have far-reaching effects. Large-scale, longitudinal studies are needed to understand how a strategic CCT system could be used to improve the health and economic conditions of families raising infants and young children in high poverty urban communities in the USA. The results of this study suggest that cash incentives to low-income parents, conditioned on investing in the human capital of their children, appear to nudge parents toward participation in programs designed to strengthen parenting without diminishing their intrinsic motivations to be what all parents wish to be: good parents.
